# Challenging clinical presentation of Zinner syndrome

**DOI:** 10.1016/j.radcr.2022.10.006

**Published:** 2022-11-03

**Authors:** Fatos Sada, Elton Cekaj, Blerina, Saraci, Ormira Shazi, Abdallah Al-Madani, Sepideh Jahanian, Shamsun Nahar, Juna Musa, Keti Mamillo, Dijon Musliu, Murtaza Ahadi, Florim Leniqi, Tamanna Agarwal, Fjolla Hyseni, Valon Vokshi, Adam Benjamin Fink, FNU Deepali, Jeton Shatri, Sadi Bexheti

**Affiliations:** a Department of Anesthesiology and Reanimation, University Clinical Center of Kosovo, Faculty of Medicine University of Prishtina, Prishtina, Kosovo; b Department of Radiology, Regional Hospital, Durres, Albania; c Department of Radiology, Mother Teresa University Hospital Center, Tirana, Albania; d Department of Radiology, Regional Hospital, Shkoder, Albania; e Internship Medical Student KHMC, Amman, Jordan; f Department of Anesthesiology, Mayo Clinic, Rochester, MN, USA; g Dhaka Medical College, Dhaka, Bangladesh; h Department of Endocrinoly Diabetes and Nutrition, Mayo Clinic, Rochester, MN, USA; i Department of Anesthesiology and Critical Care, Mother Teresa University Hospital Center, Tirana, Albania; j Department of Anatomy, Faculty of Medicine, University of Prishtina, str. Rruga e Ilindenit pa nr. 1200 Tetovë Republic of North Macedonia, Prishtina, Kosovo; k Oncology Department, Bolan Medical Complex Hospital, Quetta, Pakistan; l Faculty of Medicine, University of Gjakova, Gjakova, Kosovo; m Faculty of Medicine in Hradec Kralove, Charles University, Prague, Czech Republic; n NYU Langone Health, New York, NY, USA; o Department of Anesthesiology and Reanimation, University Clinical Center of Kosovo, Prishtina, Kosovo; p 1st Faculty of Medicine at Charles University, Prague, Czech Republic; q Department of Gastroenterology & Hepatology, Mayo Clinic, Rochester, MN, USA

**Keywords:** Zinner syndrome, Challenging clinical presentation, Role of MRI, Urology triad, Ejaculatory duct obstruction

## Abstract

Zinner syndrome is a rare congenital malformation of the mesonephric duct comprising of seminal vesicle cyst, ipsilateral renal agenesis, and ejaculatory duct obstruction. Clinical presentation varies with perineal pain, painful ejaculation, hematospermia and infertility common presenting complaints. Here, we present a case of Zinner syndrome in a 35-year-old male with a rare clinical presentation of only abdominal discomfort. The purpose of this case report is to highlight the challenging clinical presentation of Zinner syndrome and the use of imaging modalities in diagnosing the condition.

## Introduction

Zinner syndrome is a rare urological triad of unilateral renal agenesis, ipsilateral seminal vesicle cyst and ejaculatory duct obstruction. It was first reported by Zinner in 1914 [1], hence the name. It is associated with the abnormal development of the mesonephric or Wolffian duct in the first trimester of gestation [2]. Incomplete migration of the ureteric bud which originates from the Wolffian duct, causes failure to connect with the metanephros, leading to renal agenesis. However, the seminal vesicles continue to develop with insufficient drainage, resulting in distention and cyst formation [3,4]. This syndrome is mostly asymptomatic until the third or fourth decade of life, and symptoms usually manifest with the beginning of sexual activity. Patients commonly present with unspecific symptoms such as problems in voiding (dysuria, urgency, frequency), perineal pain, possible hematuria, recurrent urinary tract infections, and painful ejaculation. Infertility is also frequent, caused by ejaculatory duct obstruction [3,4]. It is an uncommon condition with less than a few hundred cases reported in literature.

## Case report

A 35-year-old man is brought from prison to the clinic with complaints of increasing abdominal discomfort. Blood pressure is 125/85 mmHg, heart rate 78/min, and temperature 37°C. Physical examination was normal. The external genitalia appear normal. There is no dysuria or hematuria. Rectal examination shows normal anal sphincter tone but a slightly enlarged prostate. PSA levels were normal. Hormonal profile and blood tests showed insignificant changes. Urinalysis was within normal range.

An abdominal ultrasound was ordered and a right cystic prominence in the urinary bladder with unique left urinary jet is revealed. Furthermore, a hypertrophied left kidney is noted.

**Fig. 1:**
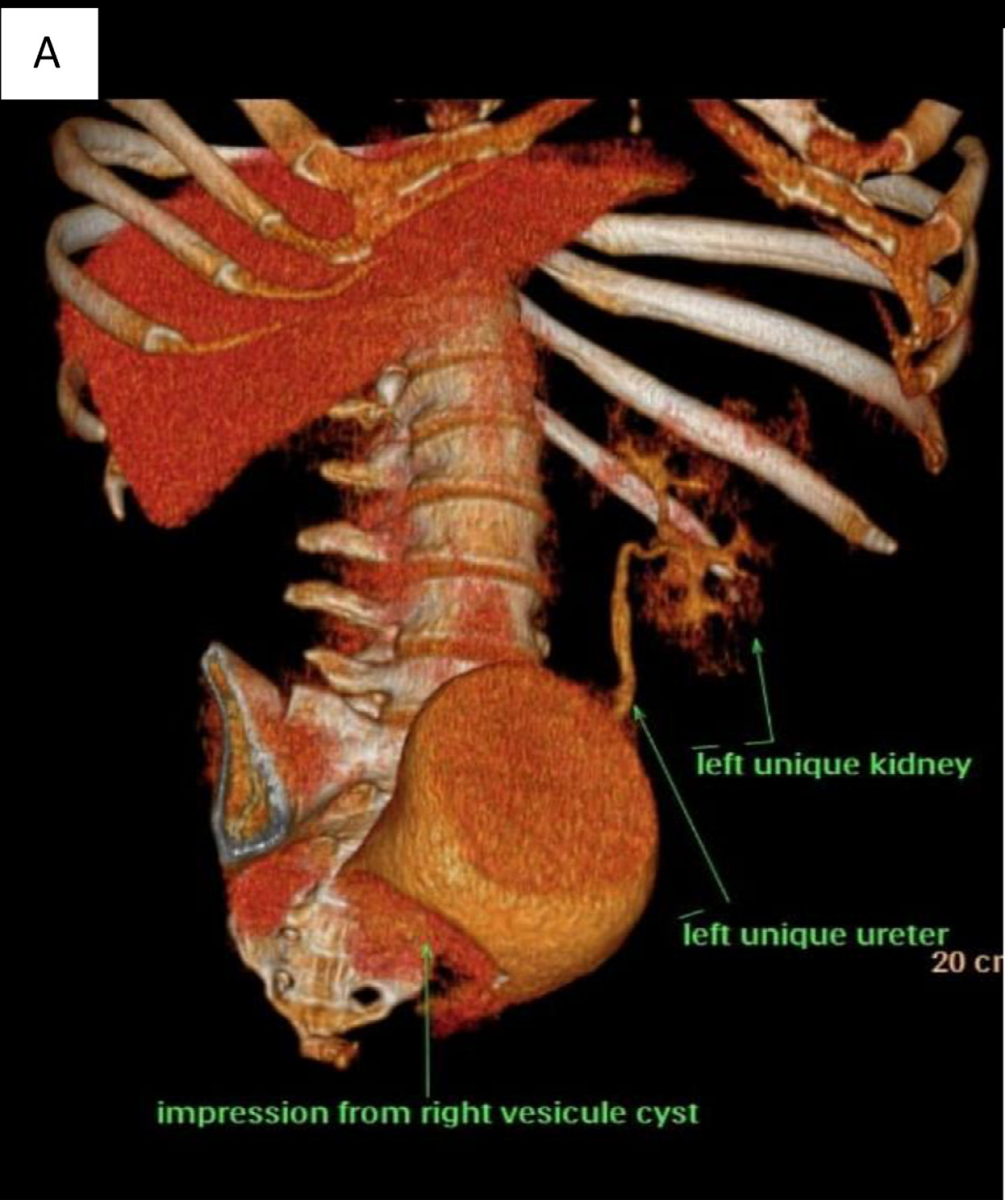

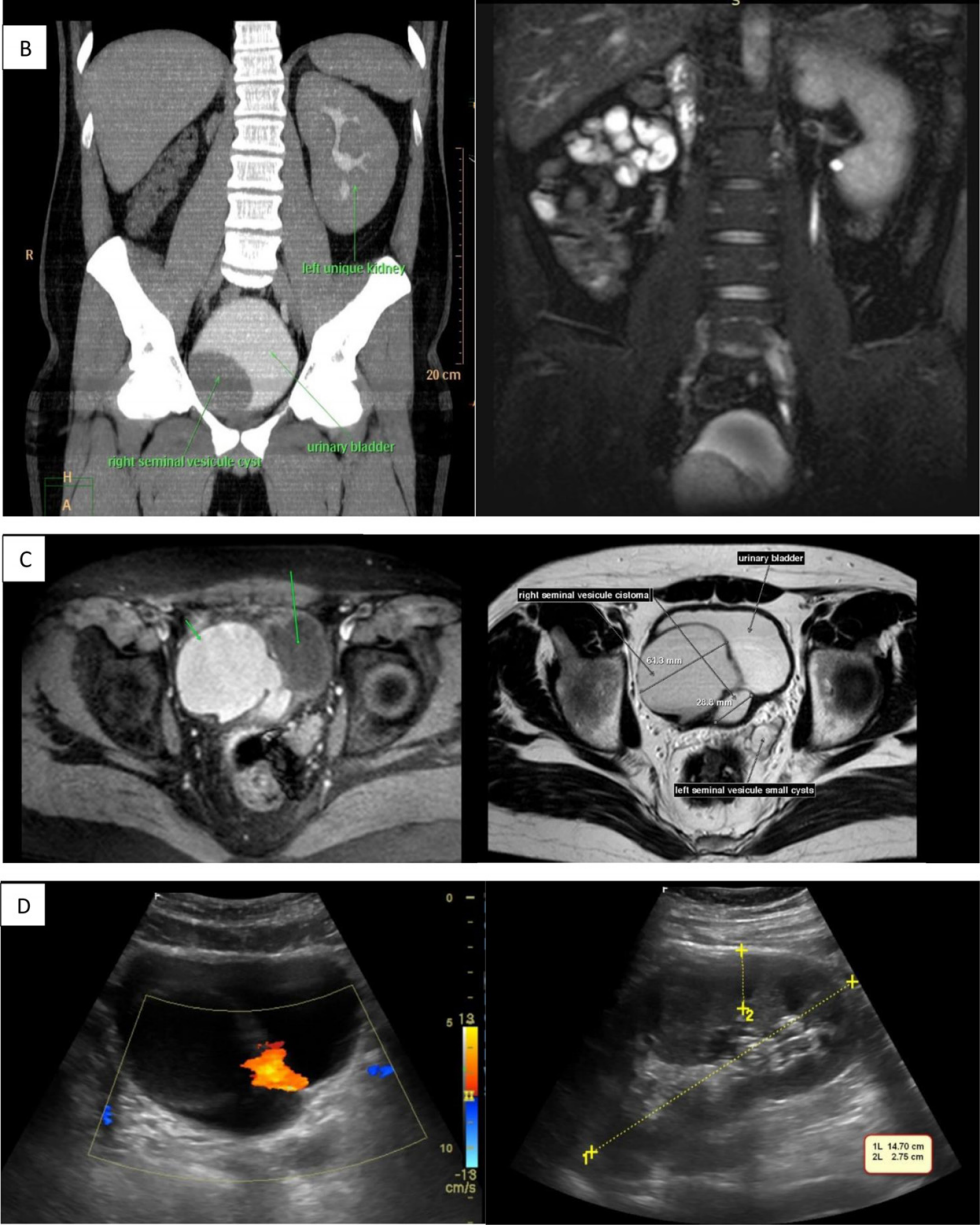
Computed tomography images (A) 3D CT: defect in the urinary bladder from right seminal vesicle cystoma and left unique kidney. (B) Coronal CT: notes the filling of the urinary bladder with contrast media and compression from the right seminal vesicle cystoma. (**C**) Axial CT: notes the filling of the urinary bladder with contrast media and compression from the right seminal vesicle cystoma. (D) Right cystoma prominence in the urinary bladder and a unique left urinary jet is revealed. Hypertrophied left kidney is noted.

Considering the ultrasound findings, Zinner syndrome was suspected. Further imaging examinations were ordered (Fig. 1).

IV contrast CT in different modes revealed:

• 3D CT: defect in the urinary bladder due to a right seminal vesicle cystoma and hypertrophied left kidney
• Axial CT: notes filling of the urinary bladder with contrast media and compression from a right seminal vesicle cystoma

Coronal CT: notes filling of the urinary bladder with contrast media and compression from a right seminal vesicle cystoma.

The MRI findings confirmed well differentiated right seminal vesicle cystoma from the urinary bladder and a hypertrophied left kidney.

Considering the imaging results, a diagnosis of Zinner syndrome was made. Surgical treatment was suggested to the patient along with close follow-up.

## Discussion

Zinner's syndrome is a triad of unilateral renal agenesis alongside ipsilateral seminal vesicle cysts and ejaculatory duct obstruction. Since Zinner first described the syndrome in 1914, approximately 100 cases have been reported in medical literature [5]. The condition originates during embryogenesis due to maldevelopment of the Wolffian duct, occurring between the 4^th^ and 13^th^ week of gestation [6]. Although it is hard to accurately determine the prevalence of the syndrome, an analysis of 280,000 children with unilateral renal agenesis conducted by Sheih et al and published in 1990, discovered six children with cystic dilations of the seminal vesicles using ultrasonography [7]. This would indicate a prevalence of 0.002142857%. MRI serves as the gold standard to make a definitive diagnosis due to its superior ability to delineate between soft tissue structures in the pelvis. Cystic dilations within the seminal vesicles are typically no larger than 5cm although 12cm dilations have been reported in the literature [5]. Patients most frequently present during their 2^nd^ and 3^rd^ decades of life [5], although about 50 pediatric cases reported in literature, with 12 of them occurring in the first year of life [8]. A systematic review analyzing 214 cases between 1999–2020 by Liu et al found that 80.8% of cases presented with clinical symptoms while the rest were discovered incidentally. The mean age of patients was 29.35 years. Furthermore, the study found that the most common clinical symptoms were dysuria (26.0%), urinary frequency (24.2%), perianal pain (20.2%), abdominal pain (14.5%), urinary urgency and infertility (13.9% each) [9].The issue of infertility is of particular importance as up to 45% of males with Zinner syndrome suffer from infertility due to ejaculatory duct obstruction. The exact pathophysiological mechanism is not fully understood as azoospermia would not be expected in the setting of a unilateral ejaculatory duct obstruction, which is part of the constitutional triad of Zinner's syndrome. Several theories have been suggested to explain how unilateral obstruction results in azoospermia and oligospermia but to date the exact mechanism is poorly understood. The first theory is that unilateral obstruction triggers an autoimmune response that produces anti-sperm antibodies that subsequently destroy sperm in the contra lateral testis. The second is that long lasting obstruction triggers the formation of reactive oxygen species that induce apoptosis in germ cells [6,10].Treatment is surgical and is offered only to symptomatic patients [6,11]. Both open and transurethral unroofing of the cysts have been done besides the more aggressive vesiculectomy [11]. Minimally invasive robotic surgery offers better overall visualization and lower injury rates [6]. For patients suffering from ejaculatory duct obstruction, trans-urethral resection of the ejaculatory duct is performed. Following surgical treatment, studies have found an improvement of semen parameters ranging from 63.0–83.0% in patients suffering from ejaculatory duct obstruction [6]. In conclusion, this case represents a less frequent yet known presentation of Zinner's syndrome, wherein the primary symptom is abdominal pain. Once a diagnosis is made utilizing correct imaging techniques, all symptomatic patients should be evaluated for surgical treatment. Additionally, they should undergo semen evaluation as surgical intervention may not only provide symptomatic relief but also improves semen parameters thus decreasing infertility.

## Conclusion

Zinner syndrome has a varied clinical presentation. Physicians and radiologists should be aware of the clinical and radiological presentation of the condition. Magnetic resonance imaging is the imaging modality of choice for diagnosing the condition. Management is aimed at pain relief with surgical intervention reserved for severe cases.

## Patient consent

We obtained written, informed consent for publication from the patient.
